# Research on railway emergency resource scheduling strategy under multiple uncertainty coupling

**DOI:** 10.1371/journal.pone.0349372

**Published:** 2026-05-19

**Authors:** Jianping Sun, Yuyang Wan, Guangle Lu, Zhaoping Tang

**Affiliations:** School of Transportation Engineering, East China Jiaotong University, Nanchang, China; Industrial University of Ho Chi Minh City, VIET NAM

## Abstract

To address the multiple sources of uncertainty in railway emergency resource scheduling, this study uses interval numbers to characterize uncertainty in scheduling time, models resource demand as a fuzzy-random variable, and integrates fuzzy credibility constraints with interval programming within a unified optimization framework. Based on interval fuzzy credibility-constrained programming, an emergency resource scheduling model is developed to minimize both scheduling time and scheduling cost. Given the characteristics of the model constructed in this paper, an improved solution strategy based on the VEPSO algorithm is designed. By using the mean of existing feasible solutions to repair infeasible solutions generated during the optimization process, the proposed method ensures that all particles satisfy the constraints. This approach effectively addresses the complex and discontinuous Pareto front characteristics inherent in multi-objective programming involving multiple uncertain variables, enabling rapid search and selection of Pareto-optimal solutions. The results of the case study indicate that the VEPSO algorithm outperforms PSO, NSGA-II, VEGA, and MOEA/D in terms of convergence efficiency and solution quality. Its average number of convergence iterations is only 385, which is approximately 26.0% lower than that of MOEA/D, with an average runtime of 273 seconds, a convergence success rate of 93.3%, and a total demand fulfillment credibility of 0.983. By introducing a satisfaction evaluation method, this study quantifies the degree of alignment between resource allocation and actual demand under different confidence levels across various accident scenarios, revealing the distribution patterns by which both factors jointly determine resource satisfaction. This provides a quantitative basis for dynamically adjusting emergency resource scheduling strategies. Furthermore, a sensitivity analysis of resource demand was conducted at a fixed confidence level. The results indicate that when the demand scaling factor k increases from 1.0 to 1.2, the rate of change in total scheduling cost is 16.0529%, and the rate of change in total scheduling time is 50%. The model exhibits a stable and approximately linear response, indicating good robustness. This study provides quantifiable decision support for railway emergency resource scheduling in environments with multiple uncertainties, offering both theoretical value and practical significance.

## 1. Introduction

As China’s railway network continues to expand and train speeds continue to increase, both the likelihood and potential consequences of railway emergencies have grown, posing serious challenges to operational safety. Railway emergencies are often characterized by strong spatial and temporal urgency, in which emergency resource scheduling is central to emergency rescue command and decision-making, and is constrained by multiple departments, operational links, and influencing factors. Investigating emergency resource scheduling strategies under multiple uncertainties can help minimize accident-related losses, improve rescue efficiency, and enhance system robustness under fluctuating conditions.

Regarding emergency resource allocation, scholars both domestically and internationally have conducted extensive research. Existing studies can be broadly categorized into two main types based on model construction: deterministic models and models under uncertainty. For deterministic models, researchers have developed multi-objective optimization models targeting parameters such as time and cost under specific scenarios, including resource shortages [1], material damage [2], and dynamic processes [3]. These models are solved using intelligent optimization algorithms like the Deep Q-Network (DQN) algorithm [4] and an improved whale optimization algorithm [5–7]. Such studies provide crucial foundational models and solution approaches for emergency resource allocation. However, in real-world emergency scenarios, parameters such as incident conditions, resource demands, and transport times are typically subject to significant uncertainty, limiting the applicability of deterministic models.

To address this limitation, methods based on uncertainty theories—including stochastic programming, fuzzy programming, and interval programming—have been successively introduced. Liu [8] addressed the issue of uncertain dispatch times during emergency resource allocation across multiple centers and incident locations by introducing the concept of fuzzy control and establishing a fuzzy stochastic programming model based on fuzzy opportunity constraints. Hu et al. [9] examined uncertainty in resource demand during railway emergencies and proposed a multi-objective scheduling model that maximized scheduling fairness and minimized transportation cost. Tang et al. [10] investigated high-speed railway emergencies under uncertain resource demand. They applied a “scenario-response” approach to estimate demand at accident points and introduced the concept of a “soft time window”. Their model minimized the total cost consisting of overtime penalty, resource transportation, and fixed scheduling of rescue points. Tan et al. [11] considered both uncertain demand at accident points and uncertain incidents in highway emergencies. They established a multi-objective optimization model with the goals of minimizing scheduling time, scheduling cost, disaster-site loss, and dissatisfaction at potential disaster locations. Li et al. [12] studied emergency resource scheduling for offshore oil spills. Considering the uncertainty of demand at spill and drift points, they adopted a robust optimization approach to construct a bi-objective model that minimized rescue cost and response time. Wang et al. [13] introduced interval numbers and triangular fuzzy numbers to describe uncertainties in resource demand, transportation time, and maximum transport capacity. They developed an emergency scheduling model under uncertain disaster information, focusing on both efficiency and fairness. Zhang [14] addressed the uncertainties and multi-stage nature of oil spill evolution in the dynamic allocation of emergency resources for major marine oil spills by constructing a three-stage fuzzy stochastic programming-based emergency resource allocation model. Li et al. [15] addressed the multi-objective optimization problem in emergency resource allocation for public health emergencies. By constructing an uncertain multi-objective programming model and employing a hierarchical sequential method for solution, they derived candidate optimal allocation schemes for emergency resources.

A comparative analysis of the aforementioned literature reveals that, although uncertainty optimization methods have been widely applied in the field of emergency resource scheduling, most existing studies focus on addressing a single type of uncertainty or, at most, a simple combination of two uncertainties. However, railway emergency resource scheduling systems typically involve multiple uncertainty factors—such as fuzziness, randomness, and interval uncertainty—concurrently. Ignoring the coupled effects of these uncertainties makes it difficult to comprehensively and accurately characterize the actual scheduling process. Furthermore, although fuzzy-stochastic programming has been attempted for such problems, it remains limited in the highly complex and incomplete context of railway emergency scenarios. Specifically, it requires the probability distributions or membership functions of uncertain parameters to be known in advance—a condition that is difficult to satisfy in the early stages of an emergency. Additionally, it tends to treat uncertainties from different sources homogeneously, making it difficult to capture their essential differences and failing to effectively incorporate decision-makers’ varying risk tolerances for different emergency objectives.

Addressing the limitations of existing research—which is largely confined to single or double uncertainty—and the shortcomings of fuzzy stochastic programming in handling complex and incomplete scenarios, this paper innovatively considers the synergistic effects of three types of uncertainty: fuzzy, interval, and stochastic. It constructs a multidimensional coupled decision-making environment that integrates scenario stochasticity, demand fuzziness, and temporal intervality, thereby overcoming the limitations of traditional models that simplify uncertainty to single or double dimensions; By integrating fuzzy credibility constraints and interval programming into a unified framework, this study pioneers a hierarchical uncertainty structure. This structure preserves interval characteristics externally while embedding fuzzy risks internally, achieving a synergistic representation where “heterogeneous uncertainties coexist without mixing,” thereby fundamentally avoiding distortion issues caused by the mixing of parameters of different natures; based on this, an improved vector-evaluated particle swarm algorithm was developed to address the multi-objective and nonlinear characteristics of the constructed model, capable of efficiently generating Pareto-optimal scheduling schemes that match different risk preferences.

The subsequent chapters of this paper are structured as follows:

Chapter 2 conducts an in-depth characterization and coupling analysis of multiple uncertainties in railway emergency resource scheduling. Chapter 3 builds upon Chapter 2 to construct an optimization model for railway emergency resource scheduling under multiple uncertainties. A fuzzy credibility constraint programming model is established and further extended into a more general interval fuzzy credibility constraint programming model.

Chapter 4 designs an improved solution strategy based on the VEPSO algorithm to address the solution requirements of the constructed model, effectively obtaining the Pareto optimal solution set for the problem.

Chapter 5 conducts validation and analysis through case studies, examining scheduling outcomes under varying confidence levels. Additionally, this chapter includes algorithm comparison experiments and sensitivity analysis of uncertain parameters to comprehensively validate the effectiveness and superiority of the proposed model and algorithm.

Chapter 6 concludes the study by summarizing the research work and key findings, while outlining future research directions.

## 2. Characterization and coupling of uncertainties

In the process of railway emergency resource dispatch, various uncertainties do not exist independently. Instead, they interact through different pathways within the same decision-making process, jointly influencing dispatch outcomes. Key elements such as scheduling time, emergency resource demand at incident locations, and incident scenarios all exhibit distinct forms of uncertainty. Their interplay results in the emergency resource dispatch problem being characterized by a distinct interweaving and coupling of multiple uncertainties.

This paper employs interval numbers to characterize the uncertainty of scheduling time, depicting the fluctuation range of transportation response times under varying operational conditions. The uncertainty of resource demand at the accident site is described using fuzzy random parameters. Its randomness stems from the unpredictability of incident scenarios, while the fuzziness of demand under a given scenario reflects the subjectivity and information incompleteness inherent in emergency resource assessment.

This study couples multiple uncertainties rather than treating them as mutually independent. They are simultaneously introduced into the model through a unified decision variable system, where they interact. The uncertainty of incident scenarios does not enter the scheduling model as independent parameters. Instead, it influences the range and membership structure of resource demand values at incident points, altering their fuzzy representation. This creates an evolutionary process where randomness and fuzziness are mutually nested. Furthermore, resource demand uncertainty participates in model construction through fuzzy credibility constraints, interacting with the interval uncertainty of dispatch timing within the same optimization framework to jointly influence dispatch decision outcomes. Consequently, the feasibility and quality of dispatch schemes depend not only on the values of individual uncertain parameters but are collectively determined by the combined effects of multiple uncertainties under the same constraints, thereby revealing the coupling characteristics among these uncertainties.

### 2.1. Uncertainty in emergency resource scheduling time

Emergency resource scheduling time refers to the maximum amount of time it takes to deliver rescue resources from the rescue point to the accident point. The operating time from the rescue point to the accident point is uncertain due to the influence of uncertain factors such as the response efficiency of the rescue point’s dispatch, the weather, the operating status of the line, and the impact of damage to the line caused by emergencies. To represent emergency resource scheduling time in an objective and realistic manner, interval numbers are used to describe the possible range of arrival times.

The road network is abstracted as an undirected weighted graph G, and the existing rescue bases, stations and accident points are selected as the vertices of the network, the arcs of the road network are the lines connecting them, and their weights are the time spent by the rescue trains running on the arcs.  G. P(P≠ϕ) is the set of all possible paths between any two points i and j, $\forall P_{L}(P_{L}\in P)$, and the traveling time via the path $P_{L}$ is denoted as an interval number ${\tilde{t}}_{L}=\left[\overset{-}{\underset{L}{t}},\overset{+}{\underset{L}{t}}\right]$ considering the uncertainty of time, where $\overset{-}{\underset{L}{t}}$ and $\overset{+}{\underset{L}{t}}$ are the lower bound (optimistic state) and the upper bound (pessimistic state) of the traveling time of the rescue train via the path $P_{L}$, respectively.

### 2.2. Uncertainty in resource demand at the accident point

Due to the uncertainty of the location of the emergency, the scale of the accident and the level of the emergency, it is difficult for the decision makers to accurately judge and predict the demand for emergency resources for each accident point, which is somewhat ambiguous. In addition, the scenarios of emergencies will also affect the demand for resources at the accident points, showing a certain degree of randomness. Therefore, the emergency resource demand has fuzzy-random double uncertainty, and its intertwined and coupled mechanism is as follows.

#### 2.2.1. Fuzzy characterization of emergency resource demand.

**Fig 1:**
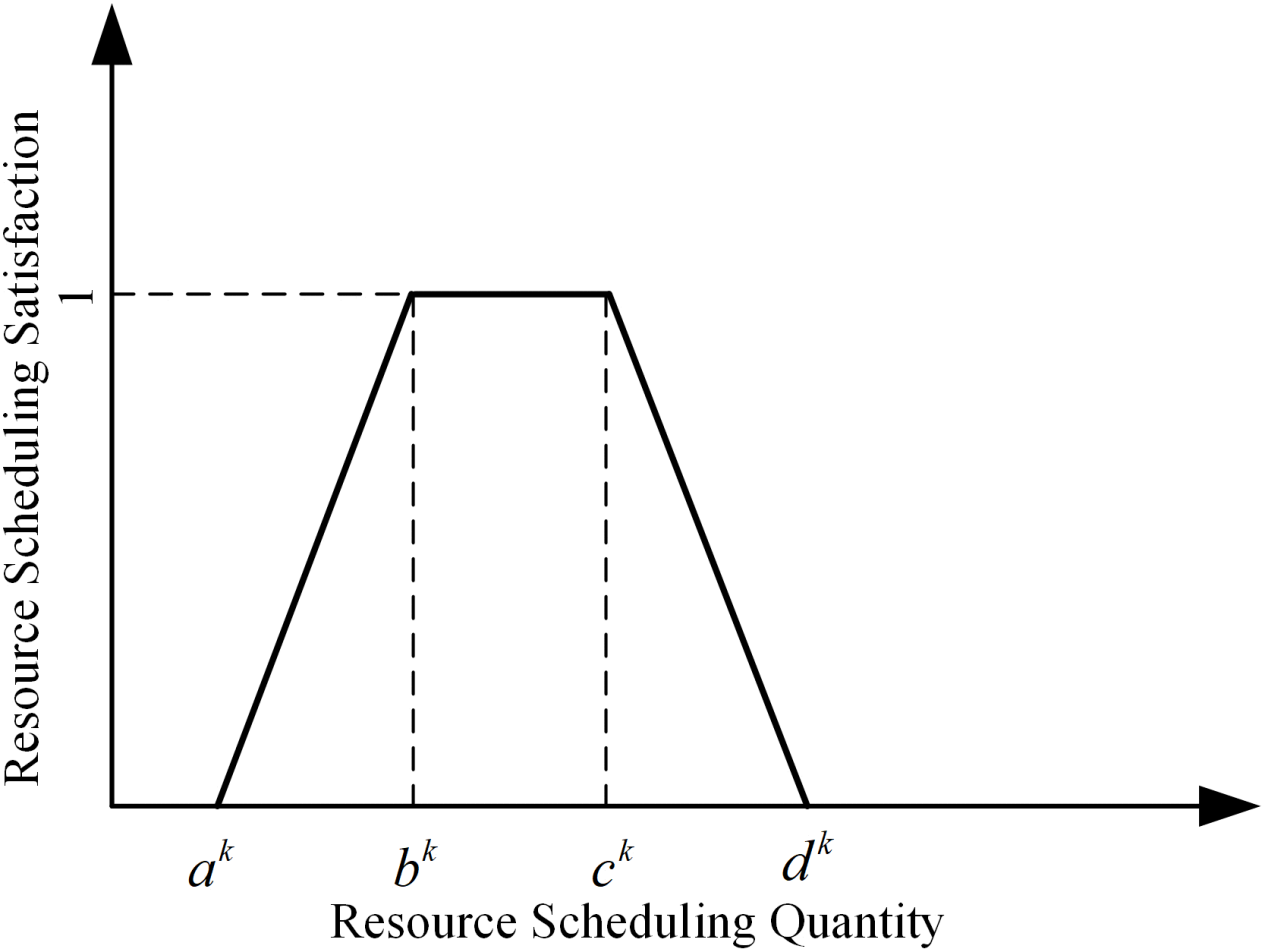
Resource scheduling satisfaction at accident point.

Considering that if the demand is too small, there will be insufficient rescue, and if the demand is too large, there will be waste or occupation of resources, trapezoidal fuzzy number is introduced to characterize the uncertainty of resource demand. The trapezoidal fuzzy number ${\tilde{Q}}^{k}=(a^{k},b^{k},c^{k},d^{k})$ represents the fuzzy demand for the kth resource at an accident point, where $a^{k}$ is the minimum demand, $b^{k}$ is the smaller value of the most likely demand, $c^{k}$ is the larger value of the most likely demand, and $d^{k}$ is the maximum demand. As shown in Fig 1, when $\;a^{k}\leq {\tilde{Q}}^{k}<b^{k}$, the satisfaction value is  (0,1), this time the function is a monotonically increasing function; when $\;b^{k}\leq {\tilde{Q}}^{k}<c^{k}$, satisfaction is 1; when $\;c^{k}\leq {\tilde{Q}}^{k}<d^{k}$, satisfaction takes the value of  (0,1), this time the function is a monotonically decreasing function. The uncertainty associated with set membership is characterized by the satisfaction degree. If the quantity of the kth type of resource received at the accident site is $\;x^{k}$, the corresponding fuzzy satisfaction degree is $\;\mu (x^{k})$, as shown in Fig 1.

#### 2.2.2. Stochastic characterization of emergency scenarios.

Due to the complexity and variety of emergency types, the severity of damage at accident point and the spreading trend of disaster all show randomness, each accident point has independent and limited scenarios. Set all scenarios of an emergency event as a set  ς, extract all the characteristic information in its life cycle, and according to the attributes and characteristics of the information content, classify the information into the event classification set $\;C=\left\{C_{\text{1}},C_{\text{2}},\cdots C_{c}\right\}$, the event severity grading $\;V=\left\{V_{\text{1}},V_{\text{2}},\cdots V_{v}\right\}$, and the disaster spreading tendency staging set $\;B=\left\{B_{\text{1}},B_{\text{2}},\cdots B_{b}\right\}$, of which  c, v and b are the number of extracted information of classification, grading, and staging characteristics in the scenario set  ς, respectively. Then, the scenario set of emergencies is $\;\varsigma =\left\{\varsigma_{\text{1}},\varsigma_{\text{2}},\cdots ,\varsigma_{\tau }\right\}$, where τ=c×v×b is the number of classified and staged feature information extracted from the scenario set. Let $\varsigma_{r}(r=1,2,\cdots \tau )$ be any scenario point in the scenario set  ς, then $\;\varsigma_{\tau }=\left\langle C_{f},V_{g},B_{\vartheta }\right\rangle$, where  f∈[1,c], g∈[1,v],  ϑ∈[1,b].

**Fig 2:**
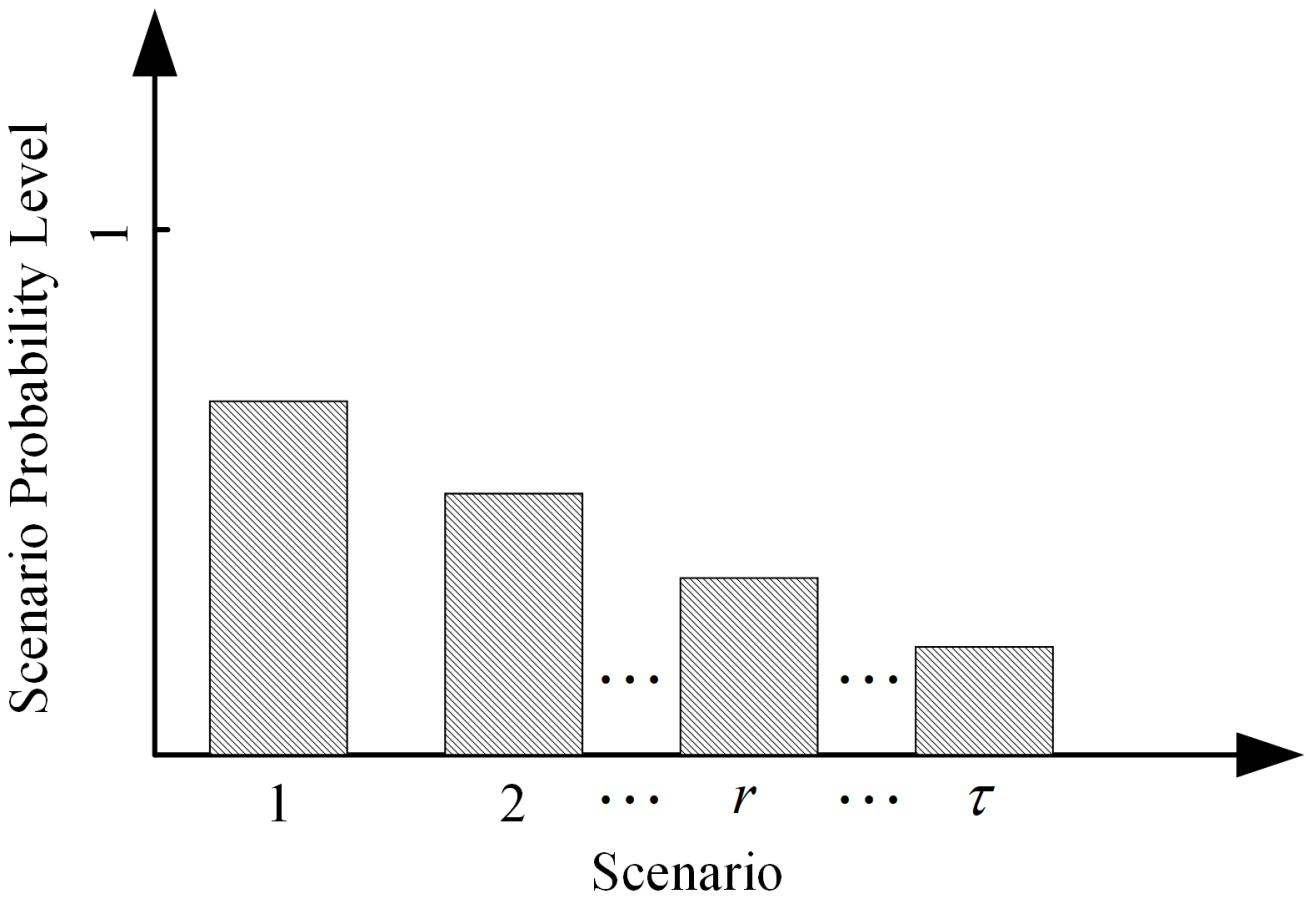
Probability level distribution under different accident scenarios.

In order to reasonably measure the stochasticity of resource demand at an accident point under different scenario levels, the likelihood of a stochastic scenario occurring can be characterized in the form of discrete probabilities, as shown in Fig 2. This figure illustrates the distribution of occurrence probability levels under different emergency incident scenarios. By abstracting all possible emergency incident scenarios into a discrete scenario set and assigning a corresponding occurrence probability to each scenario, the stochastic characteristics at the scenario level can be effectively characterized. The horizontal axis represents the accident scenario index  r(r=1,2,⋯,τ), while the vertical axis denotes the corresponding scenario occurrence probability level $\;p_{r}$. The height of each bar reflects the relative likelihood of occurrence for each accident scenario. Differences in bar heights indicate the probability weights of different scenarios within the overall set of accident scenarios, and satisfy $\sum_{r=1}^{\tau }p_{r}=1$.

#### 2.2.3. Fuzzy-stochastic coupling of emergency resource demands.

In addition to the fuzzy nature, the resource demand of each accident point is also affected by the randomness of the emergency scenarios, showing certain random fluctuation characteristics. This kind of randomness and fuzziness penetrate into each other, coupled with each other, resulting in the uncertainty of the resource demand at the accident point is no longer reflected in a single randomness or fuzziness, but presents a fuzzy-stochastic double uncertainty.

**Fig 3:**
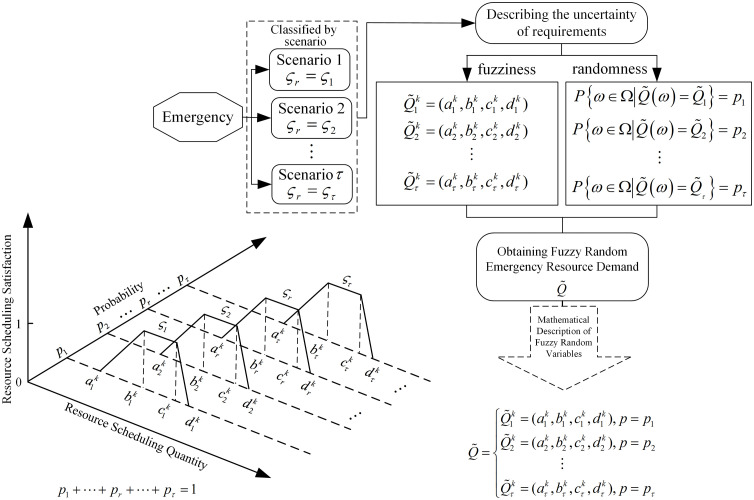
Coupling mechanism of fuzzy-random emergency resource demand.

In order to describe this phenomenon of simultaneous existence of fuzziness and randomness, the fuzzy sets whose values are taken as random quantities are portrayed, and the emergency scenarios are divided into Scenario 1, Scenario 2, …, and Scenario  τ, so as to characterize the unpredictable dynamic properties of the emergency scenarios. The trapezoidal fuzzy numbers are used to describe the range of values when the emergency resource demand is at different scenario levels, and the discrete probability form is used to characterize the possibility size of each random scenario. The coupling process between fuzziness and randomness in emergency resource demand at incident sites is illustrated in Fig 3. Accidents are categorized into discrete scenarios $\varsigma_{r}$ based on incident type, severity, and disaster propagation trends, with scenario probabilities describing their random characteristics. Under each incident scenario, resource demand at the incident site is characterized using fuzzy numbers due to incomplete information and subjective assessments. Thus, resource demand exhibits randomness at the scenario level and fuzziness within individual scenarios. Their combined effect forms a fuzzy-random demand structure. This coupling process demonstrates that the uncertainty of resource demand at the incident site cannot be described independently by a single random variable or fuzzy variable. Instead, it requires simultaneous consideration of scenario randomness and demand fuzziness within a unified framework.

According to Fig 3, it can be seen that under the random demand scenario, the satisfaction function of the accident point for the quantity of resource scheduling can be expressed as:


$$\begin{array}{c} \begin{array}{c} \mu (\overset{k}{\underset{r}{x}})=\left\{ \begin{array}{c} \frac{\overset{k}{\underset{r}{x}}-\overset{k}{\underset{r}{a}}}{\overset{k}{\underset{r}{b}}-\overset{k}{\underset{r}{a}}},\overset{k}{\underset{r}{a}}\leq \overset{k}{\underset{r}{x}}<\overset{k}{\underset{r}{b}}\\ \;1\;,\overset{k}{\underset{r}{b}}\leq \overset{k}{\underset{r}{x}}<\overset{k}{\underset{r}{c}}\\ \frac{\overset{k}{\underset{r}{d}}-\overset{k}{\underset{r}{x}}}{\overset{k}{\underset{r}{d}}-\overset{k}{\underset{r}{c}}},\overset{k}{\underset{r}{c}}\leq \overset{k}{\underset{r}{x}}<\overset{k}{\underset{r}{d}}\\ \;0\;,\;other \end{array}\right.\;\;\\ (P\left(\varsigma_{r}\right)=p_{r},r=1,2,\cdots ,\tau \;,\sum_{r=1}^{\tau }p_{r}=1) \end{array} \end{array}$$ (1)

In the formula, $\overset{k}{\underset{r}{x}}$ and $\mu \left(\overset{k}{\underset{r}{x}}\right)$ are the quantity of the kth resource and its satisfaction level under the scenario point $\;\varsigma_{r}$, respectively. Through this formula, the fuzzy resource demand at the accident point under different probability levels of emergency scenarios can be characterized as a fuzzy random variable, which realizes the measurable mapping from the probability space to the set composed of fuzzy variables.

**Fig 4:**
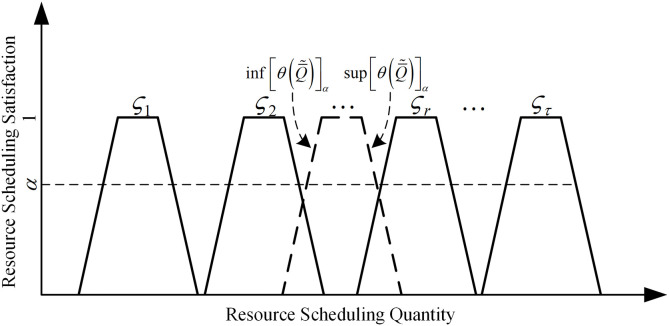
Transformation process of fuzzy-stochastic demand.


While fuzzy random variables can more accurately describe the uncertainty of resource demand, it also increases the computational difficulty of problem solving to a certain extent, therefore, using the method in the literature [16–17], the fuzzy random variable is first transformed into a trapezoidal fuzzy number during the solution process, and the computation using the fuzzy number expectation will be more efficient, and the transformation process is shown in Fig 4, ∀α∈[0,1]

$$\begin{array}{c} \inf{\left[\theta (\tilde{\overset{―}{Q}})\right]}_{\alpha }=p_{\text{1}}\inf{\left({\tilde{Q}}_{\text{1}}\right)}_{\alpha }+p_{\text{2}}\inf{\left({\tilde{Q}}_{\text{2}}\right)}_{\alpha }+\cdots +p_{\tau }\inf{\left({\tilde{Q}}_{\tau }\right)}_{\alpha } \end{array}$$ (2)

$$\begin{array}{c} \sup{\left[\theta (\tilde{\overset{―}{Q}})\right]}_{\alpha }=p_{\text{1}}\sup{\left({\tilde{Q}}_{\text{1}}\right)}_{\alpha }+p_{\text{2}}\sup{\left({\tilde{Q}}_{\text{2}}\right)}_{\alpha }+\cdots +p_{\tau }\sup{\left({\tilde{Q}}_{\tau }\right)}_{\alpha } \end{array}$$ (3)

The expectation of the fuzzy number can be obtained as:


$$\begin{array}{c} E\left[\tilde{\overset{―}{Q}}\right]=\left(\inf{\left[\theta (\tilde{\overset{―}{Q}})\right]}_{\text{0}},\inf{\left[\theta (\tilde{\overset{―}{Q}})\right]}_{\text{1}},\sup{\left[\theta (\tilde{\overset{―}{Q}})\right]}_{\text{1}},\sup{\left[\theta (\tilde{\overset{―}{Q}})\right]}_{\text{0}}\right) \end{array}$$ (4)

Figure 4 illustrates the schematic process of transforming fuzzy-random emergency resource demand into a trapezoidal fuzzy number.

It should be noted that the trapezoidal treatment of the fuzzy-random variables corresponding to Eqs. (2)–(4) is an approximately equivalent transformation for optimization purposes, rather than a lossless reconstruction of the original fuzzy-random process. This transformation preserves the main characteristics of resource demand under different accident scenarios in terms of location, core interval, and overall spread, thereby allowing the aggregated demand to participate in subsequent credibility-constrained modeling in the form of a trapezoidal fuzzy number. At the same time, however, finer-grained differences in discrete structures across scenarios, tail asymmetry, and higher-order distributional information are compressed during the aggregation process. Since the subsequent optimization model in this study mainly relies on the interval boundaries and credibility measure of fuzzy demand to determine the feasible region, rather than on higher-order statistical features of the original scenario distribution for inference, this approximate treatment can substantially reduce the computational complexity of the model while ensuring decision-making effectiveness, thereby achieving a balance between modeling accuracy and computational efficiency.

To analyze the effect of this approximation on the model results, let the original fuzzy-random variable be denoted by $\;\tilde{\xi }(\omega )$, and let its trapezoidal fuzzy approximation be denoted by $\;{\tilde{\xi }}^{T}=(a,b,c,d)$. The deviation between the two in terms of credibility can be expressed as follows:


$$\begin{array}{c} \Delta =\sup_{x}\left|Cr\left\{g(x,\tilde{\xi })\leq 0\right\}-Cr\left\{g(x,{\tilde{\xi }}^{T})\leq 0\right\}\right| \end{array}$$ (5)

In practical applications, since trapezoidal fuzzy numbers can effectively characterize the value range of a variable and its core distribution region, the aforementioned bias typically remains within an acceptable range when the membership functions of the original fuzzy random variables vary smoothly. Furthermore, the model in this paper primarily focuses on the relative merits of scheduling schemes under uncertain conditions rather than the precise characterization of individual parameters; thus, the impact of this approximation on the final decision results is limited. Therefore, converting fuzzy random variables into trapezoidal fuzzy numbers ensures the solvability of the model while striking a reasonable balance between computational complexity and expressive accuracy.

#### 2.2.4. Lemma on coupling and the fundamental theorem of non-decomposability of couplings.

1) Lemma on Coupling

Lemma 1: Let ς be a stochastic scenario, $\tilde{Q}$ be a fuzzy demand, and [t] be an interval of time. Suppose there exists a decision variable x such that:


$$\begin{array}{c} \frac{\partial^{\text{2}}}{\partial \varsigma \partial \tilde{Q}}(Cr\{\tilde{Q}(\varsigma )\cdot [t]\cdot x\})\neq 0 \end{array}$$ (6)

This indicates that there is a coupling between ς and $\;\tilde{Q}$. Actual data from railway emergency dispatching verify that this partial derivative is non-zero, confirming the existence of an intrinsic coupling.

2) Core Theorem on the Indecomposability of the Coupling

Theorem 1: For an objective function of the form $E_{\varsigma }[Cr\{\tilde{Q}(\varsigma )\cdot [t]\cdot x\}]$, the following operations are not commutative:


$$\begin{array}{c} E_{\varsigma }[Cr\{\tilde{Q}(\varsigma )\cdot [t]\cdot x\}]\neq Cr\{E_{\varsigma }[\tilde{Q}(\varsigma )]\cdot [t]\cdot x\} \end{array}$$ (7)

Proof: Let $\;\tilde{Q}(\varsigma )=(1,\varsigma ,3)$, ς~{0.5,1.5}, [t]=[2,4].Then:


$$\begin{array}{c} The\;left\;side=0.5\times Cr\{(1,0.5,3)\cdot [2,4]\}+0.5\times Cr\{(1,1.5,3)\cdot [2,4]\}=\;0.85 \end{array}$$ (8)

$$\begin{array}{c} The\;right\;side\;=\;Cr\{(1,1,3)\cdot [2,4]\}=\;0.5 \end{array}$$ (9)

Therefore, the two sides are not equal, which completes the proof. This counterexample directly demonstrates that scenario randomness and demand uncertainty must be modeled in a nested manner rather than as independent variables added together; this is the fundamental basis for the construction of an optimization model for railway emergency resource scheduling under multiple uncertainties, distinguishing it from traditional methods.

## 3. Optimization model construction of railway emergency resource scheduling under multiple uncertainties

### 3.1. Problem description

When an unexpected accident occurs in a region of the road network, it is often necessary to quickly deploy emergency resources from multiple rescue bases to meet the needs of the accident, i.e., to formulate corresponding resource scheduling programs in different emergency rescue bases. The types and quantities of emergency resources reserved by each rescue base vary according to the importance of the nodes in the network where they are located, and the cost of emergency rescue scheduling mainly includes the basic cost of emergency response of rescue bases and the cost of resource scheduling. Assuming that an unexpected accident occurs at R in a certain area of the railway network, it can be rescued by n rescue bases $\;A_{\text{1}},A_{\text{2}},\cdots ,A_{n}$, and for the k(k=1,2,⋯,K) type of emergency resources, the reserve quantity of the rescue base $A_{i}$ is $\;\overset{k}{\underset{i}{S}}$. When an accident point makes a rescue request, the decision maker, under the influence of multiple uncertainties of the emergency event, how to determine the type and quantity of resources to be dispatched from each rescue point, so that the scheduling plan can take into account the fairness and efficiency, and realize the goal of minimizing the scheduling cost and the shortest scheduling time.

### 3.2 Definition of relevant symbols and variables and model assumptions

#### 3.2.1 Definition of relevant symbols and variables.

$\overset{k}{\underset{i}{x}}$ represents the actual scheduling quantity of the kth resource transported from rescue base $A_{i}$ to accident point  R. The demand for the kth resource at accident point R is represented by the fuzzy-random parameter $\;{\tilde{Q}}^{k}=\left(a^{k},b^{k},c^{k},d^{k}\right)$. The resource scheduling time from rescue base $A_{i}$ to accident point R is represented by the interval parameter $\;{\tilde{t}}_{i}=\left[\overset{-}{\underset{i}{t}},\overset{+}{\underset{i}{t}}\right]$. At the same time, introduce the 0–1 variable, $\alpha_{i}=1$ indicates that the rescue base $A_{i}$ provided at least one emergency resource to the accident point  R, and $\alpha_{i}=0$ indicates that no resources were provided. $\lambda_{i}$ represents the credibility confidence level; $l_{i}$ represents the transportation distance from each rescue base to the accident point. $c_{i}$ represents the transportation cost per unit mileage, and ${c^{\prime }}_{i}$ is the basic start-up cost of rescue base $\;A_{i}$.

#### 3.2.2 Model assumptions.

In the initial stage of emergency response, to improve computational efficiency and highlight key resource scheduling decisions, it is assumed that each rescue train performs only one transport task within a single scheduling period. In actual operations, however, a train may complete multiple transport tasks through multiple rounds of scheduling. Therefore, this assumption is mainly applicable to the rapid deployment of resources during the initial emergency stage. At this stage, the primary objectives of decision-making are to shorten response time and achieve the rapid delivery of emergency resources. Accordingly, the model focuses on capturing the effects of multi-source uncertainties on scheduling decisions, while more complex situations involving multi-round scheduling and detailed loading and unloading operations are beyond the scope of this study.

With regard to the composition of scheduling time, this study mainly focuses on the uncertain impact of transport time. As for loading and unloading time, in railway emergency rescue scenarios, transport distance and line conditions are usually the dominant factors affecting scheduling efficiency, whereas loading and unloading time is relatively stable and accounts for only a limited proportion compared with transport time. To avoid excessive expansion of the model dimension, loading and unloading time is equivalently incorporated into the interval-valued transport time parameter for integrated representation. In this way, the solvability of the model can be maintained without altering the structure of multiple uncertainties. The specific assumptions are as follows:

1) The amount of rescue resources stored at each emergency rescue base $A_{i}$ is known as $\;S_{ik}$.
2) Each rescue train is assumed to perform one transport task in each scheduling period, with loading and unloading time equivalently incorporated into transport time. Accordingly, emergency resource scheduling time is defined as comprising response time and transport time, while the specific rescue operation time is not considered.
3) The geographic locations of the emergency rescue bases and the point of emergencies are known;
4) The base startup cost of each emergency rescue base is known.

### 3.3. Fuzzy credibility constraint programming model

For the optimization problem in which the constraint coefficients have fuzzy characteristics and are represented by the possibility distribution, the fuzzy credibility constrained programming (FCCP) method can allow the decision to meet the constraints to a certain extent, i.e., the credibility of the establishment of fuzzy constraints is greater than the pre-defined confidence level, whose expression is shown in Eq. (10).


$$\begin{array}{c} \begin{array}{c} max\;f=\sum_{j=1}^{n}c_{j}x_{j}\\ s.t.\;Cr\{\sum_{j=1}^{n}a_{ij}x_{j}\leq {\tilde{b}}_{i}\}\geq \lambda_{i}\\ \;x_{j}\geq 0,j=1,2,\cdots ,n \end{array} \end{array}$$ (10)

In the formula, $x_{j}(j=1,2,\cdots ,n)$ is the decision variable, $c_{j}$ is the profit coefficient, $a_{ij}$ is the technical coefficient, and ${\tilde{b}}_{i}$ is the fuzzy coefficient. $C_{r}$ is the credibility measure, and $\lambda_{i}$ is the credibility confidence level.

Let ξ be the fuzzy variable of membership function  μ, and r be a real number. The credibility measure is defined as follows:


$$\begin{array}{c} \;Cr\left\{\xi \leq r\right\}=\frac{\text{1}}{\text{2}}(\sup_{x\leq r}\;\mu (x)+1-\sup_{x>r}\;\mu (x)) \end{array}$$ (11)

Due to $\;Pos\left\{\xi \leq r\right\}=\sup_{x\leq r}\;\mu (x)$ and $\;Nec\left\{\xi \leq r\right\}=1-\sup_{x>r}\;\mu (x)$, the reliable measure can be transformed into:


$$\begin{array}{c} Cr\left\{\xi \leq r\right\}=\frac{\text{1}}{\text{2}}(Pos\left\{\xi \leq r\right\}+Nec\left\{\xi \leq r\right\}) \end{array}$$ (12)

Similar to the probability measure can be obtained:


$$\begin{array}{c} Cr\left\{\xi \leq r\right\}+Cr\left\{\xi >r\right\}=1 \end{array}$$ (13)

The trapezoidal fuzzy set (a,b,c,d) is chosen to characterize the fuzzy variables, and  a<b<c<d, whose affiliation function is:


$$\begin{array}{c} \mu (r)=\left\{ \begin{array}{c} @l\frac{r-b}{b-a}\;,\;\;if\;a\leq r\leq b\\ \;1\;,\;\;if\;b\leq r\leq c\\ \frac{r-c}{b-c}\;,\;\;if\;c\leq r\leq d\\ \;0\;,\;\;otherwise \end{array}\right.\; \end{array}$$ (14)

ξ≤r and the plausibility measure expression of ξ≥r is:


$$\begin{array}{c} Cr(\xi \geq r)=\left\{ \begin{array}{c} @l\;1,\;\;if\;r\leq a\\ \frac{2b-a-r}{2(b-a)},\;if\;a\leq r\leq b\\ \;\frac{\text{1}}{\text{2}},\;if\;b\leq r\leq c\\ \;\frac{d-r}{2(d-c)},\;if\;c\leq r\leq d\\ \;0,\;if\;r\geq d \end{array}\right.\; \end{array}$$ (15)

$$\begin{array}{c} Cr(\xi \leq r)=\left\{ \begin{array}{c} @l\;0,\;\;if\;r\leq a\\ \frac{r-a}{2(b-a)},\;\;if\;a\leq r\leq b\\ \;\frac{\text{1}}{\text{2}},\;\;if\;b\leq r\leq c\\ \;\frac{r-2c+d}{2(d-c)},\;if\;c\leq r\leq d\\ \;1,\;\;if\;r\geq d \end{array}\right.\; \end{array}$$ (16)

Generally, the confidence level of plausibility should be greater than 0.5, so based on the above definition of plausibility, according to the derivation of the literature [18], it can be seen that Cr(ξ≤r) and Cr(ξ≥r) can be rewritten as:


$$\begin{array}{cc} Cr(\xi \geq r)\geq \lambda & \Leftrightarrow \frac{2b-a-r}{2(b-a)}\\ \geq \lambda & \;\;\;\Leftrightarrow (2\lambda -1)a+2(1-\lambda )b\geq r \end{array}$$ (17)

$$\begin{array}{cc} Cr(\xi \leq r)\geq \lambda & \Leftrightarrow \frac{r-2c+d}{2(d-c)}\\ \geq \lambda & \Leftrightarrow 2(1-\lambda )c+(2\lambda -1)d\leq r \end{array}$$ (18)

Substituting this into the model, the fuzzy planning problem can be transformed into the following equivalent form:


$$\begin{array}{c} \begin{array}{c} min\;f=\sum_{j=1}^{n}c_{j}x_{j}\\ s.t.\;\;\sum_{j=1}^{n}a_{ij}x_{j}\leq (2\lambda_{i}-1)\overset{\text{1}}{\underset{i}{b}}+2(1-\lambda_{i})\overset{\text{2}}{\underset{i}{b}}\\ \;\;x_{j}\geq 0\;,\;i=1,2,\cdots ,n \end{array} \end{array}$$ (19)

### 3.4. Constructing an interval fuzzy credibility constrained programming model for railway emergency resource scheduling

The above method can effectively deal with the fuzzy uncertainty of the right side of the model constraints, and the other parameters are deterministic parameters. However, in the actual railway emergency rescue scheduling process there are also parameters such as emergency resource scheduling time that are expressed as interval numbers, and interval parameter planning can effectively deal with the uncertainty parameters characterized by interval numbers. This paper combines Eq. (19), innovatively integrates the interval planning model into the fuzzy credibility-constrained programming method, and constructs the interval fuzzy credibility constrained programming model (Interval Fuzzy Credibility-Constrained Programming, IFCCP), as shown in Eq. (20).


$$\begin{array}{c} \begin{array}{c} min\;f^{\pm }=\sum_{j=1}^{n}\overset{\pm }{\underset{j}{c}}\overset{\pm }{\underset{j}{x}}\\ s.t.\;\;\sum_{j=1}^{n}\overset{\pm }{\underset{ij}{a}}\overset{\pm }{\underset{j}{x}}\leq (2\lambda_{i}-1)\overset{\text{1}}{\underset{i}{b}}+2(1-\lambda_{i})\overset{\text{2}}{\underset{i}{b}}\\ \;\;x_{j}\geq 0\;,\;i=1,2,\cdots ,n \end{array} \end{array}$$ (20)

Where “-” and “+” denote the upper and lower bounds of the interval parameters, respectively.

Theorem 2: The Reduction Relationship Between IFCCP and Traditional Models

Proof:

(1) When the time interval is reduced to a fixed value, IFCCP reduces to fuzzy credibility constraint programming;
(2) When the fuzzy demand is reduced to a deterministic distribution, IFCCP reduces to interval programming;

Conversely, however, existing models cannot express IFCCP equivalently without introducing additional assumptions. This indicates that IFCCP is a generalized, non-trivial extension of the two aforementioned model classes, rather than a simple combination.

From a theoretical perspective, the IFCCP model represents a significant breakthrough over traditional fuzzy stochastic programming and interval programming models. First, there is a fundamental shift in modeling logic: IFCCP no longer attempts to convert intervals into fuzzy numbers or simplify fuzzy numbers into intervals, but instead constructs a hierarchical uncertainty structure—the outer layer retains the time-varying domain of interval parameters to avoid homogenization distortion of time parameters; the inner layer utilizes fuzzy credibility constraints to handle demand fluctuations under stochastic scenarios. This modeling approach, in which different types of uncertainty coexist without mixing, achieves a fundamental logical leap from “single-dimensional approximation”to“multi-dimensional coupled fidelity,” representing a structural innovation not found in existing methods. Second, the theoretical basis for its irreplaceability: In scenarios characterized by the triple coupling of “scenario randomness, demand fuzziness, and temporal intervality,” the sole use of fuzzy stochastic programming or interval programming inevitably leads to two types of systematic biases: one arising from approximating intervals as fuzzy numbers, and the other from simplifying fuzzy demands into intervals. For example, treating the interval parameter of rescue time as a triangular fuzzy number would render the interpretation of time-window constraint satisfaction invalid; conversely, simplifying fuzzy demands under random scenarios into intervals would result in the loss of probability weight information across different scenarios, causing high-risk, low-probability scenarios to be treated equally with low-risk, high-probability scenarios—a situation that contradicts the practical requirements of risk grading in railway emergency rescue.

In summary, the IFCCP model is currently one of the few modeling frameworks capable of simultaneously avoiding both types of bias. The IFCCP model possesses a rigorous theoretical advantage only when a problem exhibits all three characteristics: “discrete randomness of scenarios, cognitive fuzziness of requirements, and interval nature of time parameters.”

Combining the IFCCP method, considering the multiple uncertainty factors in the process of railway emergency rescue, and taking the shortest emergency rescue time and the lowest emergency rescue scheduling cost as the optimization objectives, the optimization model of railway emergency resource schedule under multiple uncertainties is established as Eqs. (21)–(29).


$$\begin{array}{c} \min\;f_{\text{1}}=\sum_{i=1}^{n}{\tilde{t}}_{i}\alpha_{i} \end{array}$$ (21)

$$\begin{array}{c} \min\;f_{\text{2}}=\sum_{i=1}^{n}l_{i}\cdot x_{i}\cdot \alpha_{i}\cdot c_{i}+\sum_{i=1}^{n}\alpha_{i}\cdot {c^{\prime }}_{i} \end{array}$$ (22)

$$\begin{array}{c} s.t.\;Cr\left\{\sum_{i=1}^{n}\overset{k}{\underset{i}{x}}\alpha_{i}\geq {\tilde{Q}}^{k}\right\}\geq \lambda^{\pm } \end{array}$$ (23)

$$\begin{array}{c} \sum_{i=1}^{n}\overset{k}{\underset{i}{x}}\alpha_{i}\leq \overset{k}{\underset{i}{S}} \end{array}$$ (24)

$$\begin{array}{c} \sum_{i=1}^{n}\alpha_{i}\geq 1 \end{array}$$ (25)

$$\begin{array}{c} \alpha_{i}=0\;or\;1 \end{array}$$ (26)

$$\begin{array}{c} \overset{k}{\underset{i}{x}}\geq 0 \end{array}$$ (27)

$$\begin{array}{c} \tilde{t}\geq 0 \end{array}$$ (28)

$$\begin{array}{c} \overset{k}{\underset{i}{x}}\in Z \end{array}$$ (29)

In the model: Eq. (21) is the objective function of the shortest emergency rescue time. Eq. (22) is the objective function of the minimum emergency rescue scheduling cost. Eq. (23) indicates that the total amount of emergency resources actually transported to the accident point by the rescuing point is not less than the fuzzy constraints of the demand of the accident point. Eq. (24) indicates that the total amount of resources provided by the rescuing point does not exceed the amount of its own storage of the resources. Eq. (25) indicates that it is guaranteed that each accident point has at least one out rescue point to provide rescue for it. Eq. (26) is a 0–1 integer constraint. Eqs. (27) and (28) are non-negative constraints. and Eq. (29) denotes that the amount of resource scheduling is rounded.

Among them, linear weighting can be utilized to deal with the interval parameters in the objective function (21), as shown in Eq. (30).


$$\begin{array}{c} t_{i}=(1-\eta )\cdot \overset{-}{\underset{i}{t}}+\eta \cdot \overset{+}{\underset{i}{t}} \end{array}$$ (30)

In Eq. (23), $\lambda^{\pm }$ is the confidence level of plausibility, and the constraints of the confidence level in the model are the resource demand quantity constraints for the accident point. When deals with simple problems, the macro decision-making can be based on the needs of decision makers to consider converting the constraints to the resource demand constraints corresponding to the accident point, and then relax the constraints to obtain a more relaxed decision-making program. The significance of confidence level in the model is shown in Table 1.

**Table 1:** Confidence level and its significance.

Confidence level	Satisfaction Level
λ=1	Fully satisfied
λ=0.9	Mostly satisfied
λ=0.8	Mostly satisfied
λ=0.7	Comparatively satisfied
λ=0.6	Barely satisfied
λ≤0.5	Does not satisfy the constraint

In summary, by coupling interval parameters with credibility constraints, the IFCCP model achieves a hierarchical characterization and unified representation of multi-source heterogeneous uncertainty. This approach not only avoids the loss of information resulting from the homogenization of uncertainty in traditional methods but also improves the accuracy of modeling complex emergency decision-making problems while maintaining the model’s solvability.

## 4. VEPSO algorithm design

For multi-objective optimization problems, traditional methods commonly adopt the weighted sum approach, in which multiple objectives are combined linearly through predefined weight coefficients, thereby transforming the problem into a single-objective optimization task. However, this method has notable limitations: the choice of weight coefficients heavily affects the results, and the single-objective optimization process often yields only local optima. Without sufficient prior knowledge, it is difficult to effectively evaluate the optimality of the solutions.

The Vector Evaluated Particle Swarm Optimization (VEPSO) algorithm [19] is a swarm intelligence-based iterative approach inspired by the concept and main ideas of the VEGA algorithm. As a co-evolutionary technique, it updates particle velocity and position iteratively to search for optimal solutions, thereby reducing the negative impact of weight settings on optimization results. The core idea of VEPSO is to divide the population into equally sized subgroups according to the number of objective functions. Each subgroup optimizes a single objective independently, while information exchange and mutual influence are realized through velocity updates of particles across subgroups [20].

However, since the model in this paper incorporates both interval uncertainty constraints and fuzzy credibility constraints, and the decision variables exhibit a mixed discrete-continuous nature, conventional VEPSO struggles to be directly applied in particle encoding, constraint handling, and feasibility checking. This may generate invalid solutions during the search process. Therefore, while retaining the multi-subpopulation cooperative search framework of VEPSO, this paper appropriately modifies its constraint handling mechanism to address the structural characteristics of the model’s constraints, thereby constructing a solution strategy suitable for the multi-uncertainty coupled scheduling model. Due to the presence of constraint (23), a dynamic repair strategy guided by feasible solutions is designed and integrated to address the complex feasible domain formed by fuzzy credibility constraints and interval constraints in the model. The core operation of this strategy is as follows: After each generation completes its iteration, the algorithm identifies all particles violating the fuzzy credibility constraints. Based on the positional statistics of feasible solutions within the current population, it applies a directed adjustment to the violating solutions using the average value of existing feasible solutions, enabling them to rapidly return to the feasible region. This repair process is adaptive and iterative. Assuming the first h particles in the swarm satisfy the constraints, if the h+1th member fails to meet the constraints, then:


$$\begin{array}{c} \left\{ \begin{array}{c} \overset{h+1}{\underset{r}{x}}=\frac{\overset{h+1}{\underset{c}{x}}+y}{\text{2}}\\ y=\frac{\text{1}}{h}\sum_{l=1}^{h}x^{l} \end{array}\right.\; \end{array}$$ (31)

Where $\overset{h+1}{\underset{c}{x}}$ represents the current value of the (h+1) th particle in the swarm. $\overset{h+1}{\underset{r}{x}}$ is the repair value. y is the average value of the h th feasible solutions. Repeat the repair process until the (h+1) th particle satisfies all the constraints. After the repair process, all the individual particles satisfy the constraints.

Under conditions of multiple coupled uncertainties and constraints, the feasible region of a scheduling problem exhibits nonlinear and contracting characteristics. If traditional penalty function methods are used to handle infeasible solutions, particles may oscillate near the boundary of the feasible region, or the search direction may deviate due to improper setting of the penalty factor, thereby reducing convergence

efficiency. The recovery strategy based on the mean of feasible solutions proposed in this paper can essentially be regarded as a statistical centering mechanism. When a particle generates an infeasible solution after updating, it is adjusted by using the mean vector of the current population’s feasible solutions as a reference point, thereby moving it toward the interior of the feasible region. Let the current set of feasible solutions be $\;F=\{x^{\left(1\right)},x^{\left(2\right)},\ldots ,x^{\left(m\right)}\}$, and its mean vector be:


$$\begin{array}{c} \overset{―}{x}=\frac{\text{1}}{m}\sum_{j=1}^{m}x^{\left(j\right)} \end{array}$$ (32)

When particle $x^{new}$ does not satisfy the constraints, Eq. (33) is used to contract the repaired solution toward the statistical center of the feasible region.


$$\begin{array}{c} x^{repair}=\alpha x^{new}+(1-\alpha )\overset{―}{x},\text{\textbackslash{}hspace\{1em}\}0<\alpha <1 \end{array}$$ (33)

The repair strategy based on the mean of feasible solutions employs a convex combination mechanism to perform a contraction mapping on infeasible solutions. This approach reduces constraint violations while enhancing search stability, and avoids the parameter sensitivity issues associated with penalty function methods as well as the boundary effects of traditional repair operators, thereby demonstrating superior overall performance in complex constrained optimization problems.

The velocity and position update equations are as follows:


$$\begin{array}{c} \left\{ \begin{array}{c} @l@\overset{j}{\underset{i}{v}}=\omega \times \overset{j-1}{\underset{i}{v}}+c_{\text{1}}\times r\times \left(\overset{j-1}{\underset{best}{p}}-\overset{j-1}{\underset{i}{p}}\right)+\\ \;c_{\text{2}}\times r\times \left(\overset{j-1}{\underset{best}{g}}-\overset{j-1}{\underset{i}{p}}\right)\\ \overset{j}{\underset{i}{p}}=\overset{j-1}{\underset{i}{p}}+\overset{j}{\underset{i}{v}} \end{array}\right. \end{array}$$ (34)

In the formula, j denotes the current iteration number. $v_{i}$ denotes the flight speed of particle  i. $p_{i}$ denotes the position of particle  i. $p_{best}$ denotes the individual optimal value. $g_{best}$ denotes the global optimal value. ω denotes the inertia weight coefficients. $c_{\text{1}}$ and $c_{\text{2}}$ denote the acceleration factors. And r is the random number that obeys the uniform distribution between  [0,1].

For multi-objective optimization problems, the algorithm first divides the population into multiple subgroups, where particles in each subgroup represent a set of relatively good solutions for the corresponding objective. The new generation of particles is generated by merging particles from all subgroups and selecting evolutionary individuals. Particle movement is influenced by the social interaction with particles from other subgroups, guiding them toward directions that simultaneously optimize more objectives. Ultimately, the algorithm converges to the optimal solution [21].

Taking the bi-objective optimization problem as an example, the implementation process of the algorithm is as follows:

**Step 1** Set the size of the particle swarm  m, the dimension  n=2×N×T, the maximum number of iterations $\;K_{\max}$, the inertia weight ω and the acceleration factors $c_{\text{1}}$ and $\;c_{\text{1}}$.

**Step 2** Generate the initial particles by randomly generating a particle swarm of size $N_{sp}$ in the feasible domain of decision variables. Determine whether the constraints are satisfied, if so, continue to Step 3. If not, repair the infeasible solution.

**Step 3** Evaluate all the particles in the particle cluster using the individual evaluation method of the single-objective optimization function, and form two sub-populations based on this: calculate the fitness values of all the particles based on the objective function $\;f_{\text{1}}(X)$, and select $f_{\text{1}}(X)$ better particles from them to form sub-particle cluster 1. Similarly, calculate the fitness values of all the particles based on the objective function $\;f_{\text{2}}(X)$, and select $\frac{N_{sp}}{\text{2}}$ better particles from them to form sub-particle cluster 2.

**Step 4** Determine the individual optimal value based on the fitness values, and determine the best particles in particle cluster 1 and particle cluster 2 respectively, which will be used as the social sharing information in the whole particle cluster.

**Step 5** For the particles in different sub-particle clusters, all of them use the social shared information from other particle clusters to adjust their flight speeds, when calculating the flight speeds of all particles in sub-particle cluster 1, the best particle information in sub-particle cluster 2 is used as the global best position Gbest1 obtained by each of the particles. Similarly, when calculating the flight speeds of all particles in sub-particle cluster 2, the best particle information in sub-particle cluster 1 is used as its best position Gbest2 obtained by each of the particles. and update the particle position and velocity information according to Eq. (34), recombine all the particles after updating to form a new particle swarm, and repair infeasible solutions in the swarm

**Step 6** Repeat Steps 3–5 until the termination condition (reaching the set accuracy or number of operations) is satisfied, and select a set of optimal solutions from the 2 sub-particle swarms to constitute the optimal solution set, which is output as the optimization result. The algorithm then terminates.

**Fig 5:**
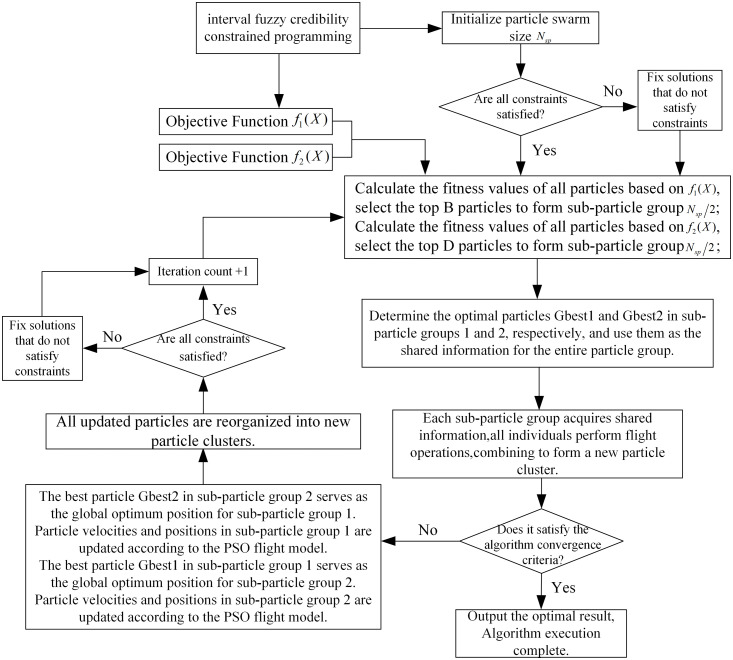
VEPSO Algorithmic framework.

The basic framework of the algorithm is shown in Fig 5.

## 5. Empirical analysis

### 5.1. Problem description

China Railway Nanchang Bureau Group Corporation (hereinafter referred to as Nanchang Bureau) is a subsidiary company of China Railway Corporation in East China. Nanchang Bureau mainly operates railway transportation and its related industries in all of Jiangxi and Fujian Provinces and part of Hunan and Hubei Provinces, occupying an extremely important position in the “eight vertical and eight horizontal” road network skeleton of the national railway. In order to cope with railway emergencies and ensure smooth railway transportation, Nanchang Bureau has established 14 rescue bases under its jurisdiction, which are respectively set up in Shangrao, Jiujiang, Yingtan, Pingxiang, Laizhou, Jingdezhen, Ganzhou, Xinyu, Fuzhou, Shaowu, Yongan, Xiamen, Xiangtang, Zhangping, and each of the bases is configured with a rescue train and related rescue materials. Assuming that a natural disaster occurs between Zixi Railway Station and Guangze Railway Station R in the area under the jurisdiction of Nanchang Bureau due to the influence of bad weather, the facilities along the line are seriously damaged, and the cars of the passing trains are damaged, with different degrees of casualties and losses, according to the principle of proximity, rescue work can be carried out by the three rescue bases in the vicinity of Shangrao $\;A_{\text{1}}$, Laizhou $\;A_{\text{2}}$, and Xiangtang $\;A_{\text{3}}$, and the emergency response resources are based on the line repairing equipment (e.g., steel rails, sleepers, etc.)$\;k_{\text{1}}$, Medical rescue equipment (e.g., medical supplies, disinfection supplies, etc.) $k_{\text{2}}$ and railway recovery equipment (e.g., rigging, spreaders, etc.) $k_{\text{3}}$ as examples for scheduling.

### 5.2. Definition of deterministic and uncertainty variables

According to the degree of impact of the accident on the personal safety situation, the number of direct economic losses, the number of passenger and freight trains derailed, and the interruption of the busy route travel time, etc., the level of sudden accidents on the railway can be classified into four different levels: especially major accidents, major accidents, relatively major accidents, and general accidents. Since the first time of the sudden accident scenario level cannot be determined, it is assumed that the probability of the appearance of each scenario is 0.1, 0.2, 0.3 and 0.4 respectively. The emergency rescue scheduling time is mainly related to the distance from the emergency rescue base to the accident point  l, the transportation speed v as well as the response time, and this paper uses the average speed of the rescue on the way to v=120km/h for the measurement. Table 2 shows the reserve quantity of emergency resources of each rescue base $\;\overset{k}{\underset{i}{S}}$, and the information between each rescue base and the accident point is shown in Table 3, which includes the transportation distance between each rescue base and the accident point  l, transportation time $\;{\tilde{t}}_{i}$, unit transportation cost of emergency resources $c_{i}$ and start-up base cost of each rescue base $\;c_{i}\prime$, where the transportation time is the number of intervals.

**Table 2:** Emergency resource inventory at rescue bases.

rescue bases	Emergency resource inventory $\;\overset{k}{\underset{i}{S}}$ at rescue base $\;A_{i}$/standard quantity		
$k_{1}$	$k_{2}$	$k_{3}$
$A_{\text{1}}$	26	13	10
$A_{\text{2}}$	15	17	18
$A_{\text{3}}$	20	10	15

**Table 3:** Information between emergency rescue bases and accident point.

	Distance between each rescue base and accident point/m	Transportation time/hour	Unit cost/yuan/standard volume/kilometer	Start-up cost/million yuan
$A_{\text{1}}$	205955	[1.5,2]	8	1
$A_{\text{2}}$	220267	[2,3]	15	3
$A_{\text{3}}$	214684	[1.5,2.5]	11	2

Due to the difficulty emergency responders face in promptly and accurately obtaining critical information, such as the affected area, casualties, and on-site conditions, during the initial stages of sudden incidents, the demand for various emergency resources at the incident site exhibits significant uncertainty. Based on the analysis in Section 2.2, this paper employs fuzzy-random variables to characterize the emergency resource requirements at the incident site.

Regarding the determination of fuzzy parameters, the emergency resource demand adopted in this study is estimated using fuzzy estimation based on historical incident data. The data construction method draws from the application of Case-Based Reasoning to railway emergency resource demand forecasting, as previously described in [22]. This approach analyzes key attribute features of historical accident cases, such as accident type, severity level, impact scope, and environmental conditions, and calculates their structural similarity to the target accident scenario to identify relevant historical precedents. Based on this, the reasonable range of demand values for various emergency resources at the accident site is determined according to the actual usage of corresponding resources in similar cases. These values are further converted into fuzzy number parameters to characterize the uncertainty level of resource demand under a given accident scenario. The central value of the fuzzy number is determined by the weighted average of demand quantities from similar cases. Upper and lower bounds are reasonably adjusted based on the discrete characteristics of historical cases and guidance from relevant domain experts, thereby constructing a fuzzy demand description that better aligns with real-world conditions. Furthermore, Eqs. (2) to (4) are used to calculate the fuzzy expected values for the demand quantities of various emergency resources at the accident site, with the results shown in Table 4.

**Table 4:** Fuzzy-random resource demands from accident point and their fuzzy expectation.

Resource	$k_{1}$	$k_{2}$	$k_{3}$
Fuzzy expected value	[22,24,25,27]	[12,14,15,17]	[19,23,26,29]

### 5.3. Algorithm Solution

To validate the model’s effectiveness and the robustness of the scheduling schemes, this paper introduces the key parameter  λ, representing the confidence level, into the solution process. The value of λ directly reflects the decision-maker’s risk attitude when dealing with uncertainty. A higher value of λ (e.g., λ=1) indicates that the decision-maker tends to be conservative, requiring the scheduling scheme to maintain extremely high feasibility even under extremely adverse conditions; while the results are undoubtedly robust, they are typically accompanied by higher contingency costs. Conversely, a lower value of λ (e.g., λ=0.6) indicates that the decision-maker is willing to seek solutions that are more optimal in terms of cost and time within an acceptable risk range, which is more aligned with rescue scenarios characterized by resource constraints or strict time constraints.

Following common parameter settings in fuzzy programming research [23–25] and considering the risk preference tiering prevalent in emergency management practice, this study selects three representative levels of  λ: 0.6, 0.8, and 1.0 for calculation. Among these, λ=0.8 represents a balanced risk attitude and is relatively common in practice. The aim is to generate a series of alternative scheduling schemes by simulating different decision-making preferences, thereby providing decision-makers with a more comprehensive decision-making space. Subsequent case studies will compare optimization results under different λ values to reveal the patterns of how changes in confidence levels affect scheduling schemes, thereby evaluating the model’s robustness and adaptability under varying risk expectations.

This example simultaneously sets up two sub-particle groups, each targeting the optimization of emergency response time and rescue cost, respectively. The Pareto optimal solution with the minimum value of the first objective function $f_{\text{1}}(x)$ is designated as the global optimal position GBest for the second sub-particle group, while the Pareto optimal solution with the minimum value of the second objective function $f_{\text{2}}(x)$ is designated as the global optimal position GBest for the first sub-particle group. Each sub-particle swarm has a size of 30, with a maximum iteration count of 1000, an inertia weight of 0.4, ωmax set to 1.2, and an acceleration factor $\;c_{\text{1}}=c_{\text{2}}=2$. Emergency resource scheduling schemes are calculated for three scenarios with confidence levels of  λ=0.6, λ=0.8 and  λ=1.

The Pareto optimal solution set is composed of a set of solutions that simultaneously satisfy the multi-objective optimization conditions, where each solution represents a different compromise solution, and each Pareto optimal solution in the Pareto optimal solution set is an optimal solution under different objectives [26]. Applying the VEPSO algorithm to solve the arithmetic example, this solution set is searched efficiently to ensure the diversity and comprehensiveness of the solutions, and the output is obtained as a set of Pareto-optimal solution sets, each of which represents a different scheduling scheme for emergency resources. In the selection of scheduling schemes, for each confidence level, the Pareto optimal solution set obtained from the operation is selected to minimize the rescue cost, and the results of the scheduling scheme are shown in Table 5. Simultaneously, comparisons were made with conventional algorithms such as Particle Swarm Optimisation (PSO), the NSGA-II algorithm and the Vector-Evaluated Genetic Algorithm (VEGA). Furthermore, to enhance the comprehensiveness of the algorithmic comparison, the Multi-Objective Evolutionary Algorithm based on Decomposition (MOEA/D)—a representative algorithm in the field of multi-objective optimisation in recent years—was introduced for comparative analysis. In the MOEA/D algorithm, the weight vectors are generated using a uniform distribution, the neighbourhood size is set to 10, and a neighbourhood-based update strategy is employed for the evolution of solutions. Each algorithm was run 30 times, with the results are shown in Table 6.

**Table 5:** Optimal emergency resource scheduling scheme under different credibility confidence levels.

Accident point	Confidence levels	$k_{1}$ Scheduling amount			$k_{2}$ Scheduling amount			$k_{3}$ Scheduling amount		
$A_{\text{1}}$	$A_{\text{2}}$	$A_{\text{3}}$	$A_{\text{1}}$	$A_{\text{2}}$	$A_{\text{3}}$	$A_{\text{1}}$	$A_{\text{2}}$	$A_{\text{3}}$
R	λ=0.6	10	0	16	11	0	10	12	0	17
	λ=0.8	24	0	12	14	0	8	10	0	15
	λ=1	16	12	10	11	3	10	10	12	7

**Table 6:** Algorithm parameters and simulation results.

options	Population size	Control parameters	$K_{\max}$	Number of iterations	Average convergence algebra	Average time taken/second	$Cr\left\{\sum_{i=1}^{n}\overset{k}{\underset{i}{x}}\alpha_{i}\geq {\tilde{Q}}^{k}\right\}$
VEPSO	30	>$\;\begin{array}{c} \omega \min=0.4\\ \omega \max=1.2\\ c_{\text{1}}=c_{\text{2}}=2 \end{array}$	1000	28/30	385	273	0.983
PSO	30	$\begin{array}{c} \omega \min=0.4\\ \omega \max=1.2\\ c_{\text{1}}=c_{\text{2}}=2 \end{array}$	1500	20/30	890	468	0.912
NSGA-II	30	$\begin{array}{c} P_{c}=0.8\\ p_{m}=0.1 \end{array}$	2000	29/30	1320	685	0.945
VEGA	30	$\begin{array}{c} P_{c}=0.8\\ p_{m}=0.1 \end{array}$	2000	22/30	1465	643	0.901
MOEA/D	30	$\begin{array}{c} T=10\\ \delta =0.9\\ n_{r}=2 \end{array}$	1000	27/30	520	315	0.962

As shown in Table 6, the VEPSO algorithm demonstrates unique optimization characteristics. Compared to the PSO algorithm, VEPSO significantly enhances search efficiency, reducing the number of convergence iterations by 59.7% while increasing the convergence success rate by 26.6%, highlighting the superiority of its algorithmic structure. Furthermore, while maintaining a high convergence success rate, the VEPSO algorithm demonstrates significant efficiency advantages. Its computational time is only 39.8% of NSGA-II, and the obtained solutions exhibit superior performance in satisfying fuzzy constraints, achieving a total demand fulfillment credibility of 0.983, a 57.5% improvement over VEGA.

A comparison with the MOEA/D algorithm indicates that MOEA/D possesses certain advantages in terms of convergence stability and solution diversity. However, in the multi-uncertainty coupled model developed in this paper, due to the complex constraint structure and the contracting nature of the feasible region, MOEA/D is slightly less efficient than VEPSO in the search for feasible solutions; its average number of convergence iterations and computational time are both higher than those of the VEPSO algorithm. In terms of solution quality, the credibility of demand satisfaction for the solutions obtained by VEPSO reached 0.983, which is higher than the 0.962 achieved by MOEA/D, indicating that VEPSO possesses a stronger ability to satisfy constraints when dealing with fuzzy credibility constraints.

These results indicate that through rational algorithmic design, VEPSO achieves a favorable balance between computational complexity and search efficiency, making it well-suited for addressing the multi-uncertainty optimization problems established in this paper. Its unique vector evaluation mechanism demonstrates distinct advantages when handling optimization problems with credibility constraints.

To evaluate the overall performance of each algorithm across multiple experiments, this paper further employs the Friedman test to conduct a multi-group comparative analysis of all algorithms. The Friedman test is a non-parametric statistical method used for comparing multiple algorithms; it ranks the performance of different algorithms across multiple experiments, calculates their average rank, and thereby determines whether there are significant differences between the algorithms.

In this study, based on the results of 30 independent experiments, the performance of each algorithm was ranked in terms of convergence rate and demand satisfaction credibility, and the average rank was calculated; the results are shown in Table 7.

**Table 7:** Results of the Friedman test of median ranks.

Average ranking	Algorithm	Rank
1	VEPSO	1.45
2	MOEA/D	2.23
3	NSGA-II	3.35
4	PSO	3.95
5	VEGA	4.17

As can be seen from Table 7, the VEPSO algorithm has the lowest average rank, indicating that it exhibits the best overall performance across multiple experiments. Furthermore, the results of the Friedman test indicate that the differences between the algorithms are statistically significant. These findings validate, from an overall perspective, the advantages of the VEPSO algorithm proposed in this paper in terms of solution efficiency and solution quality.

### 5.4. Analysis of results

#### 5.4.1. Scheduling scheme under different confidence levels.

(1) Analysis of results when λ=0.6

As shown in Table 5, when  λ=0.6, the scheduling scheme is as follows: Rescue teams from Shangrao Base $A_{\text{1}}$ and Xiangtang Base $A_{\text{3}}$ are dispatched to respond to accident point  R. Shangrao Rescue Base $A_{\text{1}}$ supplied 10, 11, and 12 units of the three emergency resource types, respectively, to accident point  R, while Xiangtang Rescue Base $A_{\text{3}}$ provided 16, 10, and 17 units. Substituting the results into Eqs. (22) and (30), respectively, we get that the target function total scheduling time is 2.1 hours and total scheduling cost is 185917 yuan.

(2) Analysis of results when λ=0.8

when  λ=0.8, the scheduling program is as follows: Shangrao Rescue Base $A_{\text{1}}$ and Xiangtang Rescue Base $A_{\text{3}}$ will rescue the accident point  R. Shangrao Rescue Base $A_{\text{1}}$ supplied 24, 14, and 10 units of the three types of emergency resources respectively, while Xiangtang Rescue Base $A_{\text{3}}$ provided 12, 8, and 15 units of the three resource types. The entire emergency rescue process recorded a total scheduling cost of 2.3 hours and incurred a total scheduling cost of 191,740 yuan.

(3) Analysis of results when λ=1

when  λ=1, the scheduling scheme is as follows: Shangrao Rescue Base $\;A_{\text{1}}$, Laizhou Rescue Base $A_{\text{2}}$ and Xiangtang Rescue Base $A_{\text{3}}$ rescue the accident point  R. Shangrao Rescue Base $A_{\text{1}}$ supplied 16, 11, and 10 units of the three types of emergency resources respectively. Laizhou Rescue Base $A_{\text{2}}$ provided 12, 3, and 12 units of the three resource types, while Xiangtang Rescue Base $A_{\text{3}}$ delivered 10, 10, and 7 units. The entire rescue process required 3 hours, with a total scheduling cost of 273,932 yuan.

In summary, the scheduling optimization model gives a variety of scheduling schemes that meet practical requirements under different confidence levels, which proves its scientific nature and can provide support for the decision maker’s scientific decision making. When  λ=1, rescue is provided by Shangrao, Laizhou and Xiangtang rescue bases, and the overall cost is the highest. From λ=1 to  λ=0.8, as the constraints are relaxed, the scheduling scheme reduces the number of rescue bases, and the rescue is provided by Shangrao Rescue Base $A_{\text{1}}$ and Xiangtang Rescue Base $A_{\text{3}}$, which is consistent with the actual needs and the cost is gradually reduced. From λ=0.8 to  λ=0.6, the constraints are more and more relaxed, and in addition to the above changes, the amount of scheduling resources of Shangrao Rescue Base is reduced, and the amount of scheduling resources of Xiangtang Rescue Base is increased. This proves the effectiveness of using interval fuzzy credibility constrained programming.

#### 5.4.2. Satisfaction analysis of scheduling schemes under different confidence levels.

Since the above scheduling plan is based on the optimization results obtained when the accident scenario rating is still unknown at the time of the accident, using the stochastic uncertainty method and considering the probability of occurrence of the four scenarios in a comprehensive manner, however, there may be differences in the performance under each specific scenario. For this reason, once the accident rating has been assessed, the above scenarios must also be evaluated for fitness and immediate scenario modifications must be made.

The satisfaction function, as an important tool for evaluating the degree of matching between fuzzy numbers, can effectively reflect the degree of satisfaction between the actual resource scheduling quantity and the fuzzy stochastic demand quantity at the accident point. When the satisfaction level is 1, it means that the scheduling plan completely meets the demand of the accident point. When the satisfaction level is between 0 and 1, it means that the plan partially meets the demand, and the larger the value means that it meets the demand to a higher degree. When the satisfaction level is 0, it means that the plan does not meet the demand at all, and a second round of emergency resource scheduling should be carried out. By calculating the resource satisfaction under each scenario, it is possible to carry out the dynamic correction of resource scheduling strategy for the decision maker in the case of gradually determining the accident level. According to Eq. (1), the total amount of all kinds of resources dispatched from each rescue base to the accident point is substituted into the satisfaction function, and the satisfaction levels of all kinds of resources at different confidence levels in different accident level scenarios are obtained, as shown in Table 8.

**Table 8:** Multi-resource satisfaction evaluation at different confidence levels in different accident level scenarios.

Confidence levels	Scenario	Multi-resource satisfaction evaluation		
		$k_{1}$	$k_{2}$	$k_{3}$
λ=0.6	$r_{\text{1}}$	0	0	0.6
	$r_{\text{2}}$	0	0.6	0.5
	$r_{\text{3}}$	0.67	1	1
	$r_{\text{4}}$	1	1	1
λ=0.8	$r_{\text{1}}$	0.67	0	0
	$r_{\text{2}}$	0.75	0.6	0.5
	$r_{\text{3}}$	1	1	0.67
	$r_{\text{4}}$	1	1	0.5
λ=1	$r_{\text{1}}$	1	0.25	0.6
	$r_{\text{2}}$	0.25	0.2	0.5
	$r_{\text{3}}$	1	1	1
	$r_{\text{4}}$	1	1	1

When  λ=0.6, the scheduling strategy completely fails to meet the demand for resources $k_{\text{1}}$ and $k_{\text{2}}$ at the accident point in scenario $\;r_{\text{1}}$, and it can partially meet the demand for resource $\;k_{\text{3}}$, which means that secondary emergency resource scheduling should be carried out immediately, and the supply of resources $k_{\text{1}}$ and $k_{\text{2}}$ should be increased as a priority. Under scenario $\;r_{\text{2}}$, the plan does not meet the demand for resource $k_{\text{1}}$ at the accident point at all, and supplementary scheduling of the resource should be carried out immediately; under Scenario $\;r_{\text{3}}$, the plan fully meets the demand for resources $k_{\text{2}}$ and $\;k_{\text{3}}$, while also achieving a better satisfaction rate for resource $\;k_{\text{1}}$. Consequently, the overall performance is robust, suggesting that suspending the secondary scheduling mechanism could be considered. under scenario $\;r_{\text{4}}$, the satisfaction level of all resources reaches 1.0, indicating that the solution fully meets the demand for all resources, and the scheduling effect has reached an optimal state, so there is no need to carry out the subsequent supplemental scheduling of resources.

When  λ=0.8, under scenario $\;r_{\text{1}}$, the program does not satisfy the demand for resources $k_{\text{2}}$ and $\;k_{\text{3}}$, and can partially satisfy the demand for resource $\;k_{\text{1}}$, which indicates that the subsequent secondary emergency resource scheduling should prioritize increasing the supply of resources $k_{\text{1}}$ and $\;k_{\text{2}}$. Under scenario $\;r_{\text{2}}$, the program can satisfy the demand for the three types of resources, and the subsequent scheduling can consider appropriately increasing a small amount of resource replenishment. Under scenario $r_{\text{3}}$ and scenario $\;r_{\text{4}}$, the program can satisfy the demand for resources $k_{\text{1}}$ and, and $\;k_{\text{2}}$, the scheduling is optimal, and there is no need for subsequent resource supplementation.

When  λ=1, Under scenario $\;r_{\text{1}}$, the program can fully meet the demand of the accident point for the resource $\;k_{\text{1}}$, and can better meet the demand for the resources $k_{\text{2}}$ and $\;k_{\text{3}}$, of which the degree of satisfaction for the resource $k_{\text{2}}$ is relatively low, the subsequent scheduling should prioritize and consider appropriately increasing the supply of the resource $\;k_{\text{2}}$. Under scenario $\;r_{\text{2}}$, the program can better meet the demand of the accident point for the various resources, and can consider the second emergency resource scheduling. Under scenarios $r_{\text{3}}$ and $\;r_{\text{4}}$, the program can fully meet the demand for the various resources. The scheduling effect has reached the optimal state, so there is no need to carry out subsequent resource supplementation.

### 5.5 Sensitivity analysis of uncertain parameters

This section conducts sensitivity analysis on the model’s key uncertain parameters, fixing the confidence level  λ=0.8, to validate the model’s robustness. In this model, transportation time serves both as an objective function and an interval uncertainty parameter. To avoid conceptual overlap and focus on the impact of external resource environment changes on scheduling decisions, this sensitivity analysis will primarily examine how fluctuations in the critical uncertain parameter, emergency resource demand, affect optimization results. As the response variable of the objective function, changes in transportation time will serve as a key indicator for evaluating the influence of demand.

To investigate how the accuracy of emergency resource demand forecasts affects scheduling plans, the distribution parameters of demand are systematically adjusted to evaluate the responsiveness and robustness of the model’s optimization objectives. Trapezoidal fuzzy numbers (a,b,c,d) are scaled proportionally by a coefficient  k, yielding new demand parameters  (k·a,k·b,k·c,k·d). During the initial phase of railway emergencies, due to information uncertainty, a 10% to 20% deviation in resource demand estimation is a common and critical risk range. This study sets k=1.1 and k=1.2 to simulate demand scales 10% and 20% higher than the baseline scenario (k=1), respectively. The aim is to analyze the response patterns of decision objectives when actual resource demands systematically exceed expectations. The sensitivity analysis results are shown in Table 9.

**Table 9:** Sensitivity analysis of resource requirements at a fixed confidence level.

Demand scaling factor k	Total scheduling cost	Overall scheduling cost change rate Δc (%)	Total scheduling time	Overall scheduling time variation rate Δt (%)
1.0	19.1740	–	2.3	–
1.1	20.4987	6.9088	2.94	27.8260
1.2	22.2520	16.0529	3.45	50

Table 9 presents a comparison of optimization results across different demand scales. It is evident that as demand scales increase systematically, both total dispatch costs and total dispatch time exhibit a significant upward trend. When demand increases by 10%, the total dispatching cost rises from 191,740 yuan in the baseline scenario to 204,987 yuan, an increase of 6.9088%. Concurrently, the total dispatching time increases from 2.3 hours to 2.94 hours, an increase of 27.8260%. When demand increases by 20%, total dispatch costs rise to 222,520 yuan, an increase of 16.0529%, while total dispatch time increases to 3.45 hours, an increase of 50%. This change stems primarily from two factors: First, to meet higher resource demands, the model must call upon emergency resource points located farther away, directly increasing transportation costs. Second, the increased total volume of resource allocation heightened the complexity and duration of overall transportation and distribution operations.

The above analysis demonstrates that the model constructed in this paper responds clearly to changes in demand scale. Decision-makers should prioritize estimating resource requirements during the early stages of a disaster. Under conditions involving multiple uncertainties, a relatively accurate scale estimate is crucial for controlling total emergency costs, ensuring timely rescue operations, and developing reliable dispatch plans.

## 6. Conclusion

This study investigates the optimization of railway emergency resource scheduling under multiple uncertainties and proposes a systematic framework spanning problem analysis, model construction, algorithm design, and case validation.

1) The multiple uncertainties faced in the railway emergency resource scheduling system are analyzed, the uncertainty of emergency resource scheduling time is portrayed by using interval numbers, the randomness brought by the scenarios of emergencies is reasonably coupled with the fuzziness brought by the demand of emergency resources, and stochastic parameters are measurably mapped from the probability space to the set of fuzzy variables, thereby forming fuzzy-random parameters. And it is incorporated into the fuzzy credibility constraints, coupling the fuzzy credibility constraints and interval planning into the same planning system, establishing the interval fuzzy credibility constrained programming model under the unified framework, and effectively solving the optimization problem of the model where the objective function and constraints contain multiple uncertainty variables.
2) Compared with PSO, NSGA-II, and VEGA algorithms, VEPSO demonstrates superior performance across all three convergence metrics: convergence iterations, runtime, and convergence success rate. Its average convergence iteration count of 385 iterations represents a 59.7% improvement over PSO’s 890 iterations, while its average convergence time of 273 seconds is 60.1% shorter than NSGA-II’s 685 seconds, maintaining a convergence success rate of 93.3%. The algorithm achieves a total demand satisfaction credibility of 0.983, representing an improvement of approximately 4% to 9% over the comparison algorithms. This demonstrates its dual advantages in handling fuzzy constraints: both convergence speed and solution quality. Furthermore, when compared with the MOEA/D algorithm—a typical multi-objective evolutionary algorithm used in recent years—the average number of convergence generations has been reduced from 520 to 385, representing a decrease of approximately 26.0%. Computational time has been reduced by approximately 13.3%, whilst the credibility of demand satisfaction has increased from 0.962 to 0.983, further validating the effectiveness of the proposed algorithm in complex environments with uncertain constraints.
3) Regarding scheduling scheme evaluation, the confidence level λ and accident scenario r jointly determine the distribution characteristics of resource satisfaction. When  λ=0.8, the scheme effectively satisfies all resources under scenario $r_{\text{4}}$ but completely fails to meet resource $k_{\text{2}}$ demand under scenario $r_{\text{1}}$. This scenario-dependent distribution of satisfaction provides a quantitative basis for dynamic emergency decision-making, enabling rapid identification of scheme weaknesses based on real-time accident severity assessments.
4) The model exhibits strong robustness against fluctuations in the demand scaling factor  k. When k increases from 1.0 to 1.2, the variation rate of total dispatch cost remains within 16.0529%, while the variation rate of total dispatch time is 50%. This approximately linear response indicates that the model output remains predictably sensitive to input perturbations, maintaining decision stability even when demand estimates contain biases.

Building upon this static optimization framework, future research should focus on developing dynamic emergency dispatch systems. By integrating real-time disaster data and resource status feedback, a rolling decision mechanism capable of adaptive adjustments throughout incident evolution can be established. This approach will enhance the timeliness and adaptability of emergency resource allocation plans.

## Supporting information

S1 File Supplementary material. **S1 Data.** Fuzzy-random resource requirements from incident locations. **S1 Table 1.** The length of the rescue path from each emergency rescue base to the accident point. **S1 Table 2.** Nanchang Railway Bureau rescue train service scope.
(ZIP)
