# Supplementary material for: Research on railway emergency resource scheduling strategy under multiple uncertainty coupling
Source: PLoS One. 2026 May 19;21(5):e0349372. doi: 10.1371/journal.pone.0349372 (PMC13186393; doi:10.1371/journal.pone.0349372)
Supplement: S1 File — S1 Data. Fuzzy-random resource requirements from incident locations. S1 Table 1. The length of the rescue path from each emergency rescue base to the accident point. S1 Table 2. Nanchang Railway Bureau rescue train service scope. (ZIP) [file pone.0349372.s001.zip › S1 Table 2.docx]

S1 Table 2 Nanchang Railway Bureau rescue train service scope

| Location | Scope of Jurisdictional Lines | Rescue radius  （km） | Total kilometers  （km） |
| --- | --- | --- | --- |
| Pingxiang | Pingxiang - Wulidun | 70.5 | 264.6 |
|  | Pingxiang - Yichun | 65.2 |  |
|  | Liling - Chaling | 116.9 |  |
|  | Chaling - Qinglu | 12.0 |  |
| Xingyu | Xinyu - Yichun | 59.8 | 327.2 |
|  | Xinyu - Zhangjiashan | 66.0 |  |
|  | Fenyi - Wenzhu | 153.3 |  |
|  | Xinyu - Shanggao | 48.1 |  |
| Xiangtang | Xiangtang - Yongxiu | 70.2 | 629.6 |
|  | Changbei - Yongxiu | 35.7 |  |
|  | Xiangtang - Jian | 200.8 |  |
|  | Xiangtang - Dongxiang | 78.0 |  |
|  | Xiangtang- Zhangjiashan | 66.6 |  |
|  | Zhangjiashan- Shangtangjianshan | 46.3 |  |
|  | Sanjiangzhen- Jiangbiancun | 107.0 |  |
|  | Fengcheng - Luoshi | 24.6 |  |
| Yingtan | Yingtan - Hengfeng, Qianshan | 67.4 | 256.6 |
|  | Yingtan - Zixi | 79.0 |  |
|  | Yingtan - Wannian | 68.7 |  |
|  | Yingtan - Dongxiang | 41.5 |  |
| Shangrao | Shangrao - Wuyishan | 84.2 | 188.5 |
|  | Shangrao - Xintangbian | 65.3 |  |
|  | Shangrao - Hengfeng | 39.0 |  |
| Jingdezhen | Jingdezhen - Daohu | 60.8 | 194.3 |
|  | Jingdezhen - Wannian | 81.0 |  |
|  | Leping - Xiangtun | 43.4 |  |
|  | Jingdezhen-Jingdezhendong | 2.3 |  |
|  | Fuliangdianzhuanxian | 4.1 |  |
|  | Liyushandianzhuanxian | 2.7 |  |
| Jiujiang | Jiujiang - Konglong | 33.0 | 382.2 |
|  | Jiujiang - Yongxiu | 89.2 |  |
|  | Jiujiang - Yongxiu | 86 |  |
|  | Jiujiangxi - Jiujiangbei | 12.5 |  |
|  | Lushan - Xihecun | 67.0 |  |
|  | Jiujiang - Xiangyu | 94.3 |  |
| Ganzhou | Ganzhoudong - Jian | 179.4 | 573.7 |
|  | Ganzhoudong - Dingnan | 158.6 |  |
|  | Ganzhou - Changting | 155.9 |  |
|  | Jiannan - Jinggangshan | 79.8 |  |
| Fuzhou | Fuzhou - Nanpingnan | 159.8 | 511.7 |
|  | Fuzhou - Fudin | 225.7 |  |
|  | Fuzhoudong - Mawei | 21 |  |
|  | Fuzhou - Putian | 105.2 |  |
| Xiamen | Xiamen - Xinanban | 102.7 | 326.3 |
|  | Zhangzhou- Zhangzhoudong | 11.4 |  |
|  | Xiamen - Putian | 148.6 |  |
|  | Xiamen - Haicang | 48.3 |  |
|  | Xiamen - Dongdu | 15.3 |  |
| Zhangping | Haishuikeng - Xiaocuo | 244.0 | 715.2 |
|  | Zhangping - Xianshi | 138.1 |  |
|  | Zhangping - Chengkou | 54.2 |  |
|  | Zhangping - Xinanban | 78.2 |  |
|  | Zhangping - Changting | 200.7 |  |
| Yongan | Yongan - Shaxian | 75 | 153 |
|  | Yongan - Chengkou | 51.2 |  |
|  | Yikou - Jiafu | 26.8 |  |
| Laizhou | Shaxian - Shunchang | 98.0 | 275.4 |
|  | Nanpingnan - Wuyishan | 148.7 |  |
|  | Laizhou - Nanpingnan | 28.7 |  |
| Shaowu | Shaowu - Shunchang | 91.1 | 165.3 |
|  | Shaowu - Zixi | 74.2 |  |
